# Monthly Summary

**Published:** 1870-09

**Authors:** 


### MONTHLY SUMMARY.

---

*The Man who may be Expected to live Long*—.He has a proper and well proportioned stature, without, however, being too tall. Ae is rather of the middle size, and somewhat thick set. His complexion is not too florid ; at any rate, too much ruddiness in youth is seldom a sign of longevity. His hair approaches rather to the fair than to the black. His skin is strong but not rough. His head is not too big ; he has large veins in the extremities ; his shoulders are round rather than flat. His neck is not too long ; his abdomen does not project ; his hands are large but uot deeply cleft. His foot is rather thick than long ; and his legs are firm and round. He has a broad, arched chest, a strong voice, and the faculty of retaining his breath for a long time without difficulty. There is harmony in all his parts. His senses are good, but not too delicate ; his pulse is slow and regular. His stomach is excellent ; his appetite is good, and digestion easy. The joys of the table are not to him of importance ; they tune his mind to serenity, and his soul partakes in the pleasure which they communicate. He does not eat merely for the sake of eating, but each meal is an hour of daily festivity. He eats slowly, and has not too much thirst—the latter being always a sign af rapid self-consumption. He is serene, loquacious, active susceptible of joy, love, and hope, but insensible to the impressions of hatred, anger, and avarice. His passions never become violent or destructive. If ever he gives way to anger, he experiences rather a useful glow of warmth, an artificial and gentle fever, without an overflowing of the bile. He is fond also of employment, particularly calm meditation and agreeable speculations. He is an optomist, a friend to Nature and domestic felicity. He has no thirst after honor or riches, and banishes all thoughts of to-morrow.—*Hufeland. N. Y. Medical Journal.*

---

*In Hiccough.*—In the *London Lancet* is reported, by Dr. John Constable, a case of hiccough complicating pneumonia, which had resisted for days various treatment and strongly threatened death, but was immediately relieved by injection of solution of muriate of morphia into the intercostal region.—*Half-yearly Compendium Med. Sci.*

*Aconite and Muriate of Ammonia in Neuralgia.*—in the Chicago *Medical Examiner* for November, Dr. J. T. Newman confirms the usefulness of aconite and muriate of ammonia in ovarian neuralgia. The prescription he gives is

| | | |
|---|---|---|
| ℞ | Ammonia muriatis.................. | ʒ ij. |
| | Tr. aconiti ....................... | f ʒ ij. |
| | Syrup aurantii cort.................. | f ℥ viij. M |

S. Teaspoonful three times a day.

